# Levamizole enhances immune responsiveness to intra-dermal and intra-muscular hepatitis B vaccination in chronic hemodialysis patients

**DOI:** 10.1186/1476-8518-4-3

**Published:** 2006-05-30

**Authors:** Hassan Argani, Ebrahim Akhtarishojaie

**Affiliations:** 1 Division of nephrology, Modarres hospital, Shahid beheshti university of medical sciences, Tehran, Iran; 2 Drug applied research center, Tabriz university of medical sciences, Tabriz, Iran; 3 Drug applied research center, Tabriz university of medical sciences, Tabriz, Iran

## Abstract

**Background:**

Hemodialysis patient are at high risk for hepatitis B virus (HBV) infection.

Although preventive vaccination is done routinely, the response to vaccination is low in this patient population. The aim of this study was to evaluate the effect of Levamizol, an enhancer of the immune responsiveness, on different routs of vaccination, i.e., intradermal (ID) versus intramuscular (IM), in stable chronic hemodialysis patients.

**Materials and methods:**

Forty four chronic heamodialyses patient were divided into four equal groups. The first group was received 40 μg HB vaccine intramuscularly. The second group was received 20 μg HB vaccine intradermally. The third and the fourth group received 20 μg vaccine IM or ID, respectively, in three doses plus oral Levamisole (100 mg for 12 day). After one and six months from the last dose of vaccine, HBs antibody titers were measured.

**Results:**

The response rate to vaccine (HBs Antibody>10 μg/L) in the routine IM HB vaccination was low (60%). It increased to 70% with ID route. Levamisole significantly raised the response rate to 90% (P < 0.01). Also in the Levamisole groups protective HB antibody titers were maintained until the end of six months.

We conclude that HD patients must be vaccinated by ID route and addition of Levamisole. Levamisole also increases antibody maintenance.

## Background

Hepatitis B virus (HBV) infection is a worldwide health problem with increased incidence in developing countries [1-4].

Despite improvements in infection control guidelines and dialysis techniques, patients with chronic renal failure (CRF) are at increased risk for HBV infection because of their suppressed immunity and frequent exposure to blood products [5-8]. Therefore, it is suggested that all CRF patients be vaccinated against HBV [9-13]. With the routine use of hepatitis B vaccination the incidence of hepatitis B infection has been reduced significantly from 30% in 1976 to 0.05% in 1997 among patients on chronic dialysis [13-15].

The increased susceptibility to infections among these patients is due to immunodeficiency status manifested by abnormal phagocytosis, T and B-lymphocyte abnormalities, and impaired responses to T cell dependent pathogens such as hepatitis B virus. Therefore, these patients are predisposed to develop chronic hepatitis infections [16-19].

Although preventive vaccination is done routinely in patients with end stage renal failure (ESRF), antibody response to vaccination is suppressed and its level rapidly declines among patients on chronic dialysis due to the decreased immunological response [15-17].

Levamisole is an anti helminthic drug which has a property to stimulate T cell activity and enhance B lymphocyte function. Thus, it can be used for up-regulation of defective immune function in patients with CRF [7]. Vaccination via the intradermal route (ID) is considered an alternative method of vaccination which could be more effective than the conventional intramuscular (IM) rout. It is effective in inducing HBs antibody production by increasing T and B lymphocyte responsiveness, probably through facilitating a greater contact with the antigen overtime [20-22].

Recent studies have shown that ID administered HB vaccine is an effective rout to induce anti-HBs Ag serum antibodies. Furthermore, the ID administration of HB vaccine has higher clinical efficacy to induce humoral immune responses than the conventional IM route [23]. The aim of this study was to investigate the effectiveness of Levamizol in enhancing the immune response to different routs of vaccination in hemodialysis patients, as well as the effect on maintenance of the protective HBs antibody titer.

## Patients and method

In our hemodialysis center from March 2002 to February 2003, 128 stable patients end stage renal disease were dialyzed 3 times per week by low flux cellulosynthetic membrane. After excluding of the patients with history of HB vaccination, current therapy with any immunosuppressive drugs, malnutrition, recent hospitalization (during the last 3 months), and positive HBs antibody and/or Hbs antigen, 44 stable chronic hemodialysis patients recruited to the study (Table 1). None of the patients had significant co-morbid conditions such as congestive heart failure, uncontrolled diabetes mellitus or liver cirrhosis.

**Table 1:** Demographic characteristics of the patients in the four groups

	Age (years)	Gender (male/female)	Underlying disease (cases)	p
Group A	47 ± 9.6	7/4	DM:(3), GN:(2), HTN:(2), UN:(3), O:(1)	ns.
Group B	45 ± 9.1	6/5	DM:(3), GN:(2), HTN:(2), UN:(3), O:(1)	ns.
Group C	48 ± 8.3	6/5	DM:(3), GN:(2), HTN:(2), UN:(3), O:(1)	ns.
Group D	41 ± 7.2	6/5	DM:(3), GN:(2), HTN:(2), UN:(3), O:(1)	ns.

It is non significant (ns) differences of age, gender and causes of renal failure in the four groups; DM: Diabetes Mellitus, GN: Glomerulonephritis, HTN: Hypertension, UN: Unknown causes, O: Other causes (includes: Alport syndrome in 1 case, Autosomal dominant polycystic disease in 1 case, Nephrolithiasis in 1 case and obstructive uropathy in 1 case).

After obtaining of informed consent, 20 or 40 microgram of recombinant human HB vaccine (from Heber Biotec, S.A., Havana, Cuba, brochure no. 1-8-0090-LI) was received to the patients three times; at the months 0, 1 and 6.

Each ml of the vaccine contained 20 microgram of surface antigen protein (with >95% purity).

The patients randomly divided in to four groups (Table 1):

1-Group A: 11 patients received 2 ml (40 μg) of the vaccine, which was administered as a single intramuscular injection in the deltoid muscle.

2- Group B: 11 patients received 1 ml (20 μg) of the vaccine intradermally in the ventral surface of the forearm.

3- Group C: 11 patients received 1 ml (20 μg) of HB vaccine intramuscularly, plus 100 mg Levamisole per day for duration of 12 days, from 6 days before of vaccination until 6 days after each vaccination.

4- Group D: 11 patients received 1 ml (20 μg) of the vaccine intradermally, plus 100 mg Levamisole per day for 12 day, from 6 days before of vaccination until 6 days after each vaccination.

Demographic characteristics and the etiology of renal failure distributed uniformly in the four groups (Tabale-1).

One and six months after the last dose of vaccination protocol HBs antibody titers were measured by ELISA method (DiaPlus Inc., Lot:8-112904, Italy).

HBs antibody titer more than10 IU was considered positive and the titer more than 100 IU was considered good responders to vaccination protocol [24,25].

The antibody responses in the 4 groups were compared by using X^2 ^and Mann-Withney *U *test in Statistical Package of Social Science (SPSS) version 12 (licensed to university of Greenwich at 2003). P < 0.05 was considered significant.

## Results

In the 44 stable patients male to female ratio was 25 to 19 with the mean ( ± SD) age of 45 ± 8.6 years. Three of the 44 subjects were excluded due to death (1 case in group A) or renal transplantation (2 cases; one case in group B and the other in group C) during the study period.

HB antibody titer in the group A was significantly lower than group B (28.9 ± 7 IU/L vs. 121.1 ± 82.3 IU/L, p = 0.04). Although the percent of seroconversion rate was lower in the group A than the group B (60% vs. 70%), it was not significant statistically. The antibody titers in groups C and D (IM and ID routs with using of Levamisole) were 541.1 ± 494.4 IU/L and 297.2 ± 9 IU/L (p = 0.6), respectively. The seroconversion rate, also, was similar in the both groups (90%) (Table 2). Thus, addition of Levamizol to intramuscular and intradermal vaccinations increased the antibody titers, 514.2 IU/L (p = 0.001) and 176.1 IU/L (p = 0.01), respectively. Evaluation of antibody titers 6 months after the last dose of vaccination showed that antibody concentration declined in all of the four groups, but this decrement was attenuated in the C group (IM+ Levamisole) and D group (ID+ Levamisole). Antibody titers in the A, B and C groups approximately were halved at the end of six months but it decreased only 30% in the D group.

**Table 2:** Hepatitis B antibody titers based on the rout of vaccination

HB vaccine groups	positive seroconversion rate after 1 month (antibody titer/IU)	positive seroconversion rate after 6 months (antibody titer/IU)
Group A (intra-muscular route by 40 microgram vaccine alone)	60% (28.9 ± 7) *P = 0.04	20% (16.7 ± 4)
Group B (intra-dermal route by 20 microgram vaccine alone)	70% (121.1 ± 82.3) #p = 0.005	60% (58 ± 7)
Group C (intra-muscular route by 20 microgram vaccine plus Levamisole)	90% (543.1 ± 494.4) §p = 0.008	80% (304 ± 7)
Group D (intra-dermal route by 20 microgram vaccine plus Levamisole)	90% (297.2 ± 90) €p = 0.6	80% (209 ± 46)

Seroconversion rate percent (when Ab titer was more than10 IU/L) and HB antibody titers (IU/L) one month and six months after different routs of vaccination. *Denotes comparison of antibody titer between groups A and B.

# Denotes comparison of antibody titer between groups B and D.

§Denotes comparison of antibody titer between groups A and C.

€ Denotes comparison of antibody titer between groups C and D.

Aside of the higher HB antibody titer in the C and D groups, percentage of patients who retained HB antibody titer as protective level was higher after six months of follow up; i.e. protective antibody level persisted in 80% of patients in the latter two groups, vs. 60% of patients in the B group and only in 20% of patients in the A group (Table 2).

Although mild localized pruritus or pain was explained by all of the patients in the B and D groups due to one ml of intradermal vaccination, any major side effect was not reported. Only one patient found mild generalized pruritus and another patient reported mild abdominal pain during the first week of Levamisole consumption, although both of the symptoms were relived by continuing of the drug and did not relapse at the succeeding courses of Levamisole in.

## Discussion

In our hemodialysis patients, antibody response (antibody titer more than 10 IU/L) to routine IM vaccination of hepatitis B was low (60%) after one month, but it increased to 70% with ID route. The response rate to both IM and ID routs of vaccination increased significantly up to 90% at the end of one month with presence of Levamisole. Addition of Levamisole could extend the protective level of HB antibody titer and also the percentage of responders at least until six months. By adding of Levamisole 80% of the patients found sustained protective antibody level after six months. Although the antibody titer secondary to vaccination in C group (IM rout vaccination plus Levamisole) was higher than D group (ID rout vaccination plus Levamisole) at this time, the difference did not reach significant statistically.

We suggest that hemodialysis patients vaccinated via the ID route would be better respond than the conventional IM rout, which also suggested by Theresa in 1998 [6], Pyone Keyi in 2002 [21] and Rangle in 2000 [4].

Addition of Levamisole to vaccination protocol would further enhance the immunological response. The difference between the two routes of injection and with or without taking of Levamisole supplement reached highly statistical significance (Table 2).

Our results further confirms the report of Ayli et al [7], which showed that Levamisole increases both serum antibody conversion rate and the duration that the antibody titer is maintained. Kayatas [26] in another study reported the effect of Levamisole on the percentage of patients who found HB Antibody seroconversion but they did not explain the amount of antibody titers in detail. In our study only non-vaccinated stable hemodialysis patients were received Levamisole via ID or IM routs, but in the Kayatas study response rate of the pre-vaccinated patients via conventional rout was examined. The higher antibody response after ID vaccination could be due to more effector immune cells accessible in derm composed of Langerhans and dendritic cells [27]. The difference of immunomodulatory response to vaccination routs would be disappeared when Levamisole is added to the vaccination protocol.

Quadruple injection protocol and addition of booster doses of vaccine when is necessary was suggested by Vlassopoulos DA etal. [28]. But it is not cost effective for developing countries, such as us. Each dose of vaccine is approximately U.S.$ 3 but Levamisole has negligible price with minimal side effects if it be used for a short period of time such as 12 days as we used. The plasma elimination half-life of Levamisole is between 3–4 hours. Levamisole is extensively metabolized by the liver in humans and the metabolites excreted mainly by the kidneys (70% over 3 days). The elimination half-life of metabolite excretion is 16 hours. Approximately 5% is excreted in the feces. Less than 5% is excreted unchanged in the urine and less than 0.2% in the feces. The drug is not dialyzable, by hemo- or peritoneal membranes [29].

The antibody production rate could be more stimulated by triggering of T helper cells, such as Levamisole or other immunomodulatory drugs. Levamisole can act either as immunostimulant depending upon dose administered the timing of its administration and host genetic background [30]. Levamisole also could induce an increase in the level of interleukin 2 receptor [31] and T helper to T suppressor ratio in many dermatologic disorders [32].

We conclude that hemodialysis patients should be vaccinated perfectly by ID or IM route in addition of Levamisole. Future studies with larger number of patients are needed to validate our results as a routine protocol in hemodialysis patients.
